# Conventional and synthetic MRI in multiple sclerosis: a comparative study

**DOI:** 10.1007/s00330-017-5100-9

**Published:** 2017-11-13

**Authors:** Wolfgang Krauss, Martin Gunnarsson, Margareta Nilsson, Per Thunberg

**Affiliations:** 1 Department of Radiology, Faculty of Medicine and Health, Örebro University, SE-701 85 Örebro, Sweden; 2 School of Medical Science, Faculty of Medicine and Health, Örebro University, Örebro, Sweden; 3 Department of Neurology and Neurophysiology, Faculty of Medicine and Health, Örebro University, Örebro, Sweden; 4 Faculty of Medicine and Health, Örebro University, Örebro, Sweden; 5 Centre for Medical Imaging and Physiology, Skåne University Hospital, Lund, Sweden; 6 Department of Medical Physics, Faculty of Medicine and Health, Örebro University, Örebro, Sweden

**Keywords:** Multiple sclerosis, Magnetic resonance imaging, Inter- and intra-observer agreement, Synthetic MRI, Quantitative MRI

## Abstract

**Objectives:**

To compare the assessment of patients with multiple sclerosis (MS) using synthetic and conventional MRI.

**Materials and methods:**

Synthetic and conventional axial images were prospectively acquired for 52 patients with diagnosed MS. Quantitative MRI (qMRI) was used for measuring proton density and relaxation times (T1, T2) and then, based on these parameters, synthetic T1W, T2W and FLAIR images were calculated. Image stacks were reviewed blindly, independently and in random order by two radiologists. The number and location for all lesions were documented and categorised. A combined report of lesion load and presence of contrast-enhancing lesions was compiled for each patient. Agreement was evaluated using kappa statistic.

**Results:**

There was no significant difference in lesion detection using synthetic and conventional MRI in any anatomical region or for any of the three image types. Inter- and intra-observer agreements were mainly higher (*p* < 0.05) using conventional images but there was no significant difference in any specific region or for any image type. There was no significant difference in the outcome of the combined reports.

**Conclusion:**

Synthetic MR images show potential to be used in the assessment of MS dissemination in space (DIS) despite a slightly lower inter- and intra-observer agreement compared to conventional MRI.

***Key Points*:**

*• Synthetic MR images may potentially be useful in the assessment of MS.*

*• Examination times may be shortened.*

*• Inter- and intra-observer agreement is generally higher using conventional MRI.*

## Introduction

Today there are several alternatives to quantitative MRI (qMRI) that enable concurrent measurements of the relaxation times (T1 and T2) and proton density (PD) in clinical MR systems [1–5]. These tissue parameters can be exploited for different applications including the creation of synthetic MR images and segmentation of white matter (WM), grey matter (GM) and cerebrospinal fluid (CSF).

Synthetic MR images are calculated according to mathematical expressions to give image contrasts analogous to conventional T1 and T2 weighting (T1W and T2W) as well as FLAIR [1, 5]. The synthetic images, including T1W, T2W and FLAIR, are thus obtained from one single acquisition compared to conventional imaging where image contrast series are obtained one by one.

The qMRI sequence named QRAPMASTER [1] has been implemented in different MRI systems [6, 7] together with the possibility to pursue calculation of synthetic MR images. Studies of synthetic MR imaging mimicking conventional T1W, T2W and FLAIR have shown that the image qualities of T1W and T2W are comparable but synthetically calculated FLAIR images have an inferior quality compared to conventional images [6, 8].

Multiple sclerosis (MS) is an inflammatory demyelinating disease of the central nervous system. Neurological deficits arise from focal lesions in the brain or spinal cord. MR imaging constitutes a cornerstone in MS diagnosis and for monitoring of treatment [9]. Standardised MR protocols in MS include T1W, T2W and FLAIR images [10, 11]. The evaluation of the examination involves identification of MS lesions regarding the number and localisation.

Synthetic images were in a recent study compared to conventional images in terms of MS lesion detection in order to compare diagnostic accuracy [6]. The study showed a good agreement between the numbers and volumes of identified lesions using the two alternatives.

Administration of gadolinium (Gd) contrast agent enables detection of contrast-enhanced active MS plaques in T1W images. Such highlighted areas are important in staging MS since they correspond to inflammatory processes and damage of the blood-brain barrier. The use of synthetic T1W images to identify active MS plaques has not been compared to conventional MRI even though the combination of synthetic MR and Gd contrast has been investigated in another context indicating differences when imaging before and after the administration of contrast agent [12].

The aims of this study were to compare (1) the diagnostic outcome between synthetic and conventional MRI in terms of detection of MS lesions in T1W, T2W and FLAIR images after the administration of Gd contrast agent, (2) the inter- and intra-observer agreement and (3) the lesion-to-white matter contrast and signal-to-noise ratio of the two imaging alternatives.

## Materials and methods

### Subject group

All regulatory obligations for a prospective observational study were fulfilled and approval was received from the Regional Ethics Review Board. Informed consent was obtained from all patients participating in the study. Fifty-five MS patients fulfilling the revised McDonald diagnostic criteria [13] were consecutively examined and recruited by a consultant in neurology (M.G.) between January 2011 and May 2013. Some patients have previously been reported in [14], but then however in another context.

Three patients recalled from consent. The remaining group of 52 patients consisted of 41 females and 11 males, the mean age was 42 years (range 22 – 65 years) and the mean disease duration was 12 years (range 2 – 34 years). Clinical subtypes were classified as relapsing-remitting in 43 patients, secondary-progressive in 8 patients and 1 patient had primary-progressive MS. The mean Expanded Disability Status Scale Score (EDSS) was 3.0 (range 0 – 8).

Sample size (N) was estimated to detect a difference (paired) of three lesions between two reviewers assuming a standard deviation in difference of 6. This was met for *N* = 33 with a power of 0.8 and a significance level (two-sided) of 0.05. Examinations showing confluent white matter lesions were not included in the study.

### MR examination protocol

All MR examinations were performed on a Philips Achieva 1.5-T system (Philips Medical Systems, Best, The Netherlands). An 8-channel SENSE head coil or a 16-channel SENSE neurovascular coil was used as receiver coil.

MR sequence parameters are shown in detail in Table 1. The clinical routine MR protocol for examination of the MS patients included the following sequences: axial diffusion-weighted imaging (DWI), axial and sagittal FLAIR, axial T2W fast spin echo and gadolinium-enhanced axial T1W spin echo. Gadolinium contrast agent [Omniscan (Gadodiamide, GE Healthcare, UK), 0.1 mmol/kg bodyweight] was administered to the patient after the initial DWI as a bolus intravenous injection. The qMRI sequence QRAPMASTER [1] was added last in the protocol and encompassed the whole brain with equal slice angulation, slice thickness, gap, positioning and field of view (FOV) as the conventional axial FLAIR, T1W and T2W series. QRAPMASTER is a multi-slice, multi-echo and multi-saturation recovery pulse sequence using an optional number of echo times (TE) and saturation delay times (TD).

**Table 1.** Parameters used for conventional MRI sequences, QRAPMASTER and synthetic MRI generation

Parameter	Diffusion-weighted imaging (DWI)	Conventional T1W	Conventional T2W	Conventional FLAIR	QRAPMASTER	Synthetic T1W	Synthetic T2W	Synthetic FLAIR
Image plane	Axial	Axial	Axial	Axial/sagittal	Axial	Axial	Axial	Axial
TR (ms)	3024	475	9784	11,000	4445	500	4500	12,000
TE (ms)	93	12	110	140	15/30/45/60/75	10	100	100
TI (ms)	-	-	-	2800	-	-	-	2600
TD (ms)	-	-	-	-	106/602/1992/4274	-	-	-
Acquisition matrix	112 x 89	224 × 168	352 × 264	272 × 198/272 × 189	152 × 152	152 × 152	152 × 152	152 × 152
Reconstruction matrix	128 × 128	512 × 512	512 × 512	512 × 512	-	256 × 256	256 × 256	256 × 256
Acquisition resolution (mm)	2.1 × 2.6	1 ×1.4	0.7 × 0.9	0.9 × 1.2/0.9 × 1.2	1.5 × 1.5	1.5 × 1.5	1.5 × 1.5	1.5 × 1.5
Image in-plane resolution (mm)	1.8 × 1.8	0.5 × 0.5	0.5 × 0.5	0.5 × 0.5	0.9 × 0.9	0.9 × 0.9	0.9 × 0.9	0.9 × 0.9
Field of view (mm)	230	230	230	230	230	230	230	230
Slice thickness (mm)	5	3	3	3/5	3	3	3	3
Interslice gap (mm)	1	0.3	0.3	0.3	0.3	0.3	0.3	0.3
b-Value (s/mm^2^)	0/1000	-	-	-	-	-	-	-
Number of averages	1	2	2	1/1	1	-	-	-
Echo train length	47 (EPI-factor)	1	15	27	25	-	-	-
SENSE factor	2	1	2	1.5/1	1.2	-	-	-
Scan time (min:s)	0:36	6:31	2:27	3:40/5:52	6:27	-	-	-

TR: repetition time; TE: echo time; TI: inversion time; TD: delay time

### MR image post processing

The T1 and T2 relaxation times were determined from the combination of images obtained with different TE and TD. Images acquired at different TE were used for calculation of T2 while images acquired at different TD were used for calculation of T1. A mono-exponential fit of the image data to the expression of the signal intensity in the QRAPMASTER images gave an estimate of the unsaturated magnetisation (M_0_) in each voxel, which then was rescaled to give the proton density [1, 15].

Image sets acquired with QRAPMASTER were exported to the PACS system (IDS 7, version 14.3.14.2, SECTRA Medical Systems, Linköping, Sweden) and uploaded into the SyMRI software (version 7.0.2, Synthetic MR AB, Linköping, Sweden), where the calculation of T1, T2 and PD was performed as well as the generation of the synthetic T1W, T2W and FLAIR images (see Table 1 for parameter settings). The contrast in the synthetic images was calculated using well-known mathematical equations that include both tissue (T1, T2 and PD) and pulse sequence (TR, TE and TI) parameters [1]. Default values of TR, TE and TI given in the SyMRI software were used in the calculation of the synthetic images. All images, both conventional and synthetic, were saved in PACS as image stacks for review.

### Image analysis

Conventional and synthetic axial T1W, T2W and FLAIR images were separately analysed blindly, independently and in random order in a PACS workstation by a general radiologist with 13 years of experience (W.K., denoted reviewer 1) and a subspecialist in neuroradiology with 15 years of experience (M.N., denoted reviewer 2). Repeated analysis was performed by one radiologist (W.K.) six weeks apart. Hyperintense MS lesions on T2W and FLAIR images and contrast-enhancing MS lesions on T1W images were counted. The location of each lesion was documented (juxtacortical, periventricular or infratentorial) and also summarised to give the total number of cerebral lesions. The review of all images was performed in the same PACS system and displayed on the same kind of workstation.

Cerebrum, juxtacortical and periventricular lesion counts were categorised in three groups (0-9, 10-20 and >20 lesions) based on the number of lesions found in the T2W and FLAIR images. The rationale for grouping the number of lesions is based on recommendations from the Swedish MS Society on how to report MR findings in follow-up examinations of MS patients [9]. Two categories (0 and ≥ 1 lesions) were used for grouping infratentorial T2 lesions and contrast-enhancing lesions.

A summary radiology report was created for each patient that contained: (1) the lesion counts in the cerebrum identified in the T2W images, (2) the lesion counts in the cerebrum identified in the FLAIR images and (3) the presence or non-presence of any contrast-enhancing lesion.

For comparison of lesion-to-white matter contrast and signal-to-noise ratio measurements between conventional and synthetic images, all patients with contrast-enhancing lesions on T1W images were selected. Circular regions of interest (ROIs) were placed in contrast-enhancing lesions and adjacent normal-appearing white matter in T1W, T2W and FLAIR images to assess lesion-to-white matter contrast. The signal-to-noise ratio was measured by placing ROIs in five anatomical areas (CSF, left centrum semiovale, anterior horn of the corpus callosum, left thalamus, left frontal cortex). The mean and standard deviation of the intensity for each ROI were registered. Lesion-to-white matter contrast was determined as the difference between lesion and white matter intensity divided by the white matter intensity. The signal-to-noise ratio was calculated for each ROI by dividing the mean intensity by the standard deviations.

### Statistical methods

The Shapiro-Wilk test was used to assess for the normality of distribution of lesion counts as well as values for lesion-to-white matter contrast and signal-to-noise ratio.

The differences of cerebrum lesions counts between the reviewers are presented with median and the inter-quartile range (IQR) due to non-Gaussian distribution of the data. Two-sided Wilcoxon rank sum test was used to identify any statistically significant differences.

Linear kappa was used as a measure of agreement. Inter- and intra-observer agreement was calculated using the grouped data of lesions found in T1W, T2W and FLAIR images obtained from conventional and synthetic MRI. In terms of agreement, the kappa value was interpreted as: poor < 0.20, fair 0.21-0.40, moderate 0.41-0.60, good 0.61-0.80 and very good 0.81-1.00. Confidence intervals (95% CI) of the kappa values were used to find possible non-overlapping intervals that, in this case, indicate a significant difference (*p* < 0.05). To get an overall comparison of all inter- and intra-observer agreements, a paired t-test of all kappa values was performed.

McNemar’s test was used to examine if there was any significant difference in radiology report consistency when the reviewers used conventional or synthetic images. Report consistency occurred when the two reviewers judged criteria (1), (2) and (3) similar.

Bland-Altman analysis [16, 17] with 95 % limits of agreement was used to show differences in lesion counts in conventional versus synthetic T1W, T2W and FLAIR images.

The normally distributed samples for the lesion-to-white matter contrast and signal-to-noise ratio in conventional vs. synthetic MR images were compared using a two-sided paired t-test, while for non-normally distributed samples the Wilcoxon rank sum test for paired samples was applied.

Statistical analysis was performed using the MedCalc software (MedCalc for Windows, release 16.4.1, MedCalc Software, Ostend, Belgium).

## Results

**Fig. 1.**
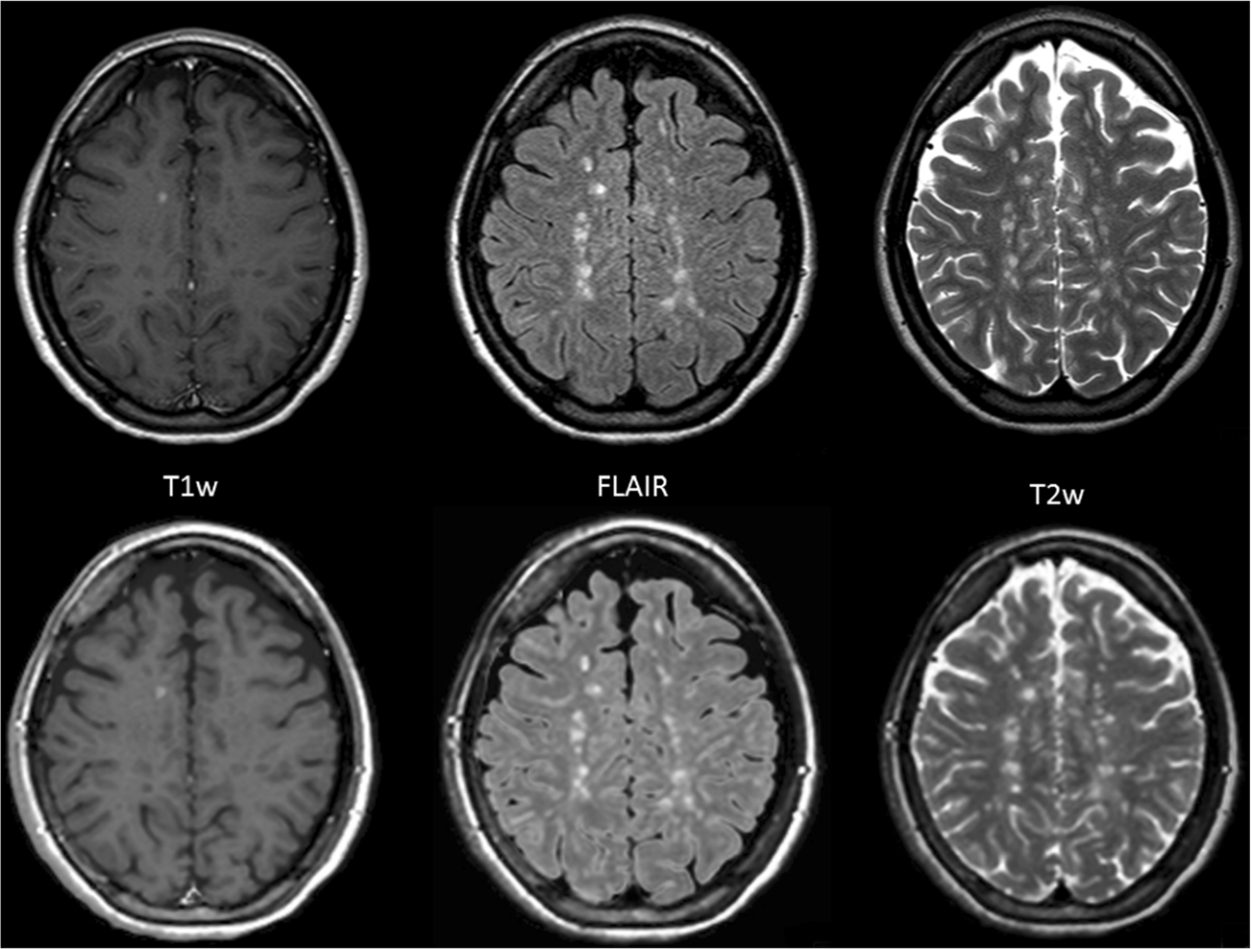
Example of conventional images (*upper row*) and synthetic T1W (contrast-enhanced), T2W, and FLAIR images (*lower row*) in an MS patient

Among the MS population, 13 individuals had confluent white matter lesions yielding 39 examinations for review of T2W and FLAIR images while all 52 included examinations contained T1W image stacks that were usable for analysis. In total, 260 image stacks were reviewed by both reviewers. Examples of conventional and synthetic images are shown in Fig. 1.

A summary of total lesion count differences in the cerebrum is presented in Table 2. There were no significant differences in lesion counts between conventional and synthetic MR for all three images types (T1W, T2W, FLAIR). The lesion detection in terms of inter- and intra-observer agreement showed similar results for conventional and synthetic images.

**Table 2.** Difference in cerebrum lesion counts between the two reviewers in T1W, T2W and FLAIR images expressed with median and the inter-quartile range (IQR) within parentheses. The 2.5 and 97.5 percentiles were used to give the range of difference for the T1W images since the IQR was equal to zero in these cases

	Conventional vs. Synthetic		Inter-observer difference		Intra-observer difference	
	Reviewer 1	Reviewer 2	Conventional	Synthetic	Conventional	Synthetic
T1W	0.0 (-2.0 – 6.2)	0.0 (-1.2 – 5.2)	0.0 (-2.0 – 2.4)	0.0 (-1.2 – 1.2)	0.0 (-1.0 – 2.4)	0.0 (0.0 – 1.0)
T2W	1.0 (-2.0 – 4.0)	1.0 (-2.0 – 3.8)	-1.0 (-3.0 – 1.8)	-2.0 (-4.8 – 1.0)	-2.0 (-5.0 – 1.8)	-2.0 (-4.0 – 1.8)
FLAIR	1.0 (-4.0 – 5.8)	-1.0 (-5.0 – 1.0 )	1.0 (-1.0 – 3.0)	-2.0 (-8.3 – 2.0)	-2.0 (-4.0 – 0.0)	-1.0 (-3.0 – 2.0)

**Fig. 2.**
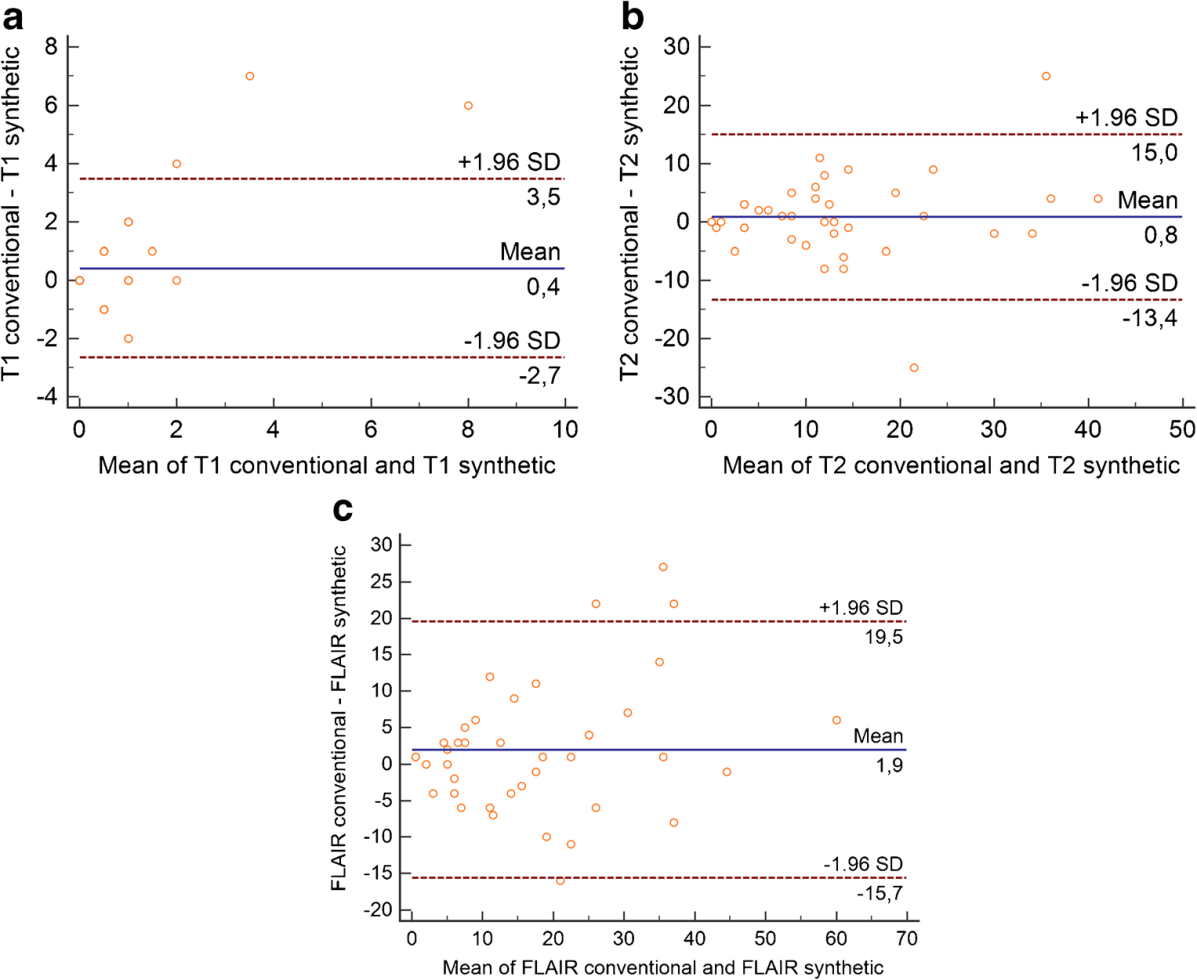
Bland-Altman plots showing the differences between the total number of detected lesions in conventional and synthetic images: (**a**) T1W, (**b**) T2W and (**c**) FLAIR images (reviewer 1)

Bland-Altman plots in Fig. 2 illustrate the differences in detected lesions as documented by reviewer 1 for the different images type. As appreciated in Fig. 2a contrast-enhancing lesions were found in ten patients; in the remaining patients no enhancing lesion was found and these data points coincide in the origin of the plot.

Agreement on lesion counts between conventional and synthetic MR did not differ significantly in any region or for any type of image since there were overlapping confidence intervals in all cases; see Table 3. In the infratentorial region, however, the inter- and intra-observer agreement was exclusively higher using conventional images compared to synthetic images. Also, the inter-observer agreement regarding the total number of identified lesions in the cerebrum was higher using conventional images for all three image types. The kappa values in Table 3 obtained for conventional images were significantly higher (*p* = 0.0285) compared to the synthetic images.

**Table 3.** Kappa values with confidence intervals (CI) for inter- and intra-observer agreement for conventional and synthetic MR images in the whole cerebrum and different anatomical regions

		Inter-observer agreement		Intra-observer agreement	
		Conventional	Synthetic	Conventional	Synthetic
Cerebrum	T1W	0.64 (CI 0.41 to 0.87) Good	0.51 (CI 0.25 to 0.78) Moderate	0.71 (CI 0.49 to 0.93) Good	0.90 (CI 0.76 to 1.00) Very good
	T2W	0.76 (CI 0.59 to 0.93) Good	0.56 (0.36 to 0.76) Moderate	0.80 (CI 0.66 to 0.94) Good	0.69 (0.52 to 0.86) Good
	FLAIR	0.77 (CI 0.62 to 0.92) Good	0.69 (CI 0.52 to 0.85) Good	0.80 (CI 0.66 to 0.94) Good	0.80 (0.66 to 0.94) Good
Periventricular	T1W	0.81 (CI 0.60 to 1) Very good	0.67 (0.38 to 0.96) Good	0.65 (CI 0.37 to 0.93) Good	0.88 (0.64 to 1.00) Very good
	T2W	0.44 (0.26 to 0.62) Moderate	0.69 (CI 0.45 to 0.92) Good	0.62 (CI 0.40 to 0.83) Good	0.46 (0.24 to 0.68) Moderate
	FLAIR	0.60 (CI 0.38 to 0.82) Moderate	0.30 (0.06 to 0.54) Fair	0.64 (0.46 to 0.82) Good	0.74 (0.53 to 0.94) Good
Juxtacortical	T1W	0.56 (CI 0.26 to 0.87) Moderate	0.91 (0.74 to 1.00) Very good	0.74 (CI 0.45 to 1.00) Good	0.84 (CI 0.62 to 1.00) Very good
	T2W	0.59 (CI 0.33-0.85) Moderate	0.35 (CI 0.01 to 0.69) Fair	0.80 (CI 0.62 to 0.98) Good	0.39 (CI 0.04 to 0.74) Fair
	FLAIR	0.80 (CI 0.65 to 0.95) Good	0.66 (CI 0.46 to 0.86) Good	0.91 (CI 0.80 to 1.00) Very good	0.72 (CI 0.50 to 0.93) Good
Infratentorial	T1W	0.79 (CI 0.39 to 1.00) Good	0.37 (0 to 0.93) Fair	1.00 (CI 1.00 to 1.00) Very good	0.79 (CI 0.39 to 1.00) Good
	T2W	0.63 (CI 0.39 to 0.88) Good	0.42 (CI 0.14 to 0.71) Moderate	0.63 (CI 0.38 to 0.88) Good	0.53 (0.26 to 0.80) Moderate
	FLAIR	0.50 (CI 0.23 to 0.77) Moderate	0.28 (CI 0 to 0.58) Fair	0.81 (CI 0.59 to 1.00) Very good	0.54 (CI 0.27 to 0.82) Moderate

The proportion of consistent radiology reports using conventional images was higher (62%) compared to synthetic images (51%) but the difference was not significant.

The lesion-to-white matter contrast was statistically significantly higher (*p* < 0.0001) in conventional contrast-enhanced T1W images while there was no significant difference in T2W (*p* = 0.34) and FLAIR (*p* = 0.50) images. Signal-to-noise ratios (see Table 4) were significantly higher in synthetic T2W and FLAIR images compared to conventional images except for CSF in T2W images where there was no significant difference. The differences in SNR between synthetic and conventional images were not consistent in T1W as in T2W and FLAIR.

**Table 4.** Differences in signal-to-noise ratio (SNR) between synthetic and conventional images in different regions. Normally distributed differences are presented as mean ± 1 SD while non-normally distributed differences are presented with median and inter-quartile range (IQR)

Anatomical region	Difference in SNR (synthetic – conventional)		
	T1W	T2W	FLAIR
Cerebrospinal fluid (CSF)	76 ± 61 **	14 ± 26	6.4 ± 3.8 **
Left centrum semiovale	3 ± 19	4.5 ± 1.7 ***	7.0 ± 6.4 *
Anterior horn of corpus callosum	-14 ± 13 *	19 ± 16 *	12 ± 8 **
Left thalamus	1.1 (-4.1 – 13.7)	6.2 (4.7 – 8.9) *	7.6 (4.3 – 8.3)**
Left frontal cortex	2 ± 16	14 ± 15*	9.9 ± 8.9 *

* p<0.05; ** p<0.01; *** p<0.001

## Discussion

The results of this study showed no significant differences in inter- and intra-observer agreement regarding detection of MS lesions using conventional and synthetic MR images, but a tendency to poorer agreement in synthetic images. No statistically significant difference between total lesion counts in synthetic vs. conventional MR images was observed. Also, a lower percentage of consistent radiology reports was observed when using synthetic MR, but the difference was not statistically significant. The lesion-to-white matter contrast was significantly higher in conventional contrast-enhanced T1W images while no difference was noticed in T2W and FLAIR images. Signal-to-noise ratios were mainly higher in synthetic T2W and FLAIR images compared to conventional images.

A limited number of studies in the literature have investigated the inter-observer agreement of conventional MR in MS. One study showed poor agreement for the total number of lesions and moderate agreement when using dichotomised composite criteria according to Barkhof, Fazekas and Paty [18] or the McDonald criteria [19]. Another study assessing the inter-observer agreement regarding dissemination in space (DIS) and dissemination in time (DIT) [20] showed moderate agreement for neuroradiologists trained in using diagnostic criteria published by the International Panel on the diagnosis of MS [13].

Some studies assessing diagnostic performance of synthetic MRI have been published. Synthetic MR images were perceived to be of inferior quality, but agreed with the clinical diagnosis (MS versus non MS) to the same extent as the conventional images [8]. A recent study performed with synthetic MR on a 3-T system showed no statistically significant differences in lesion detection and diagnostic classification between synthetic and conventional MR images. The differences in lesion counts between synthetic and conventional images were on the same order of magnitude as differences between observers [6]. In another recently published study detection of brain metastases in conventional T1W images was compared to synthetic T1W and T1 inversion recovery (T1IR) images. The study was performed on a 3-T system and obtained similar results using synthetic images compared to conventional images [7].

Our findings are in accordance with the results of these earlier studies and add information about the diagnostic performance of contrast-enhanced T1W images in MS.

**Fig. 3.**
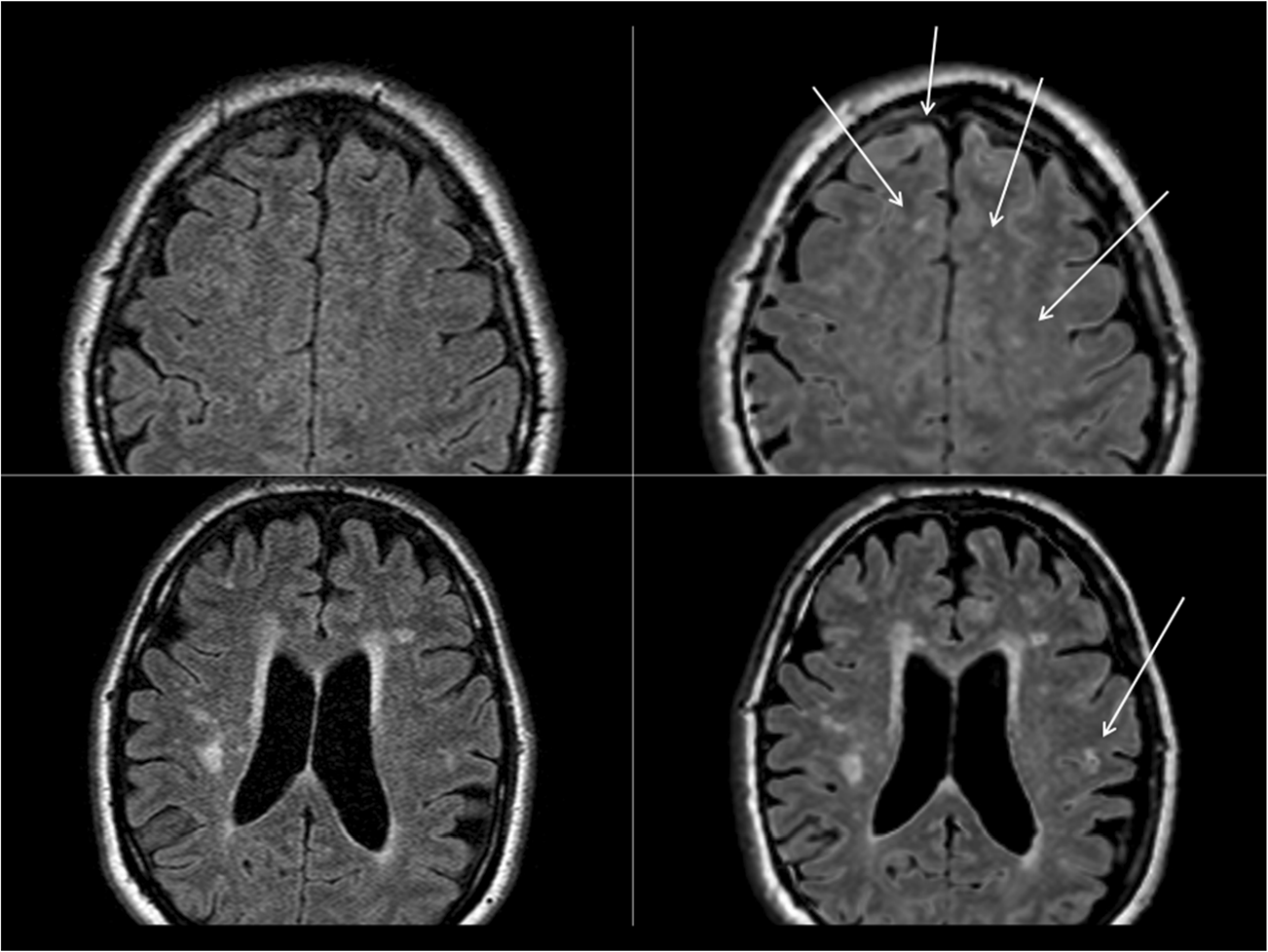
Conventional (*left column*) and synthetic (*right column*) FLAIR images. Suspicious MS lesions on synthetic images (*arrows*) are less obvious on conventional images

Variations in lesion counts could partly be explained by pulsation artefacts in T1W images. In both conventional and synthetic T2W and FLAIR images the distinction of large, focal periventricular lesions and confluating lesions may be difficult and affect lesion counts. In FLAIR images, areas with high contrast between grey and white matter and small vessels could simulate small MS lesions, particularly in the centrum semiovale (Fig. 3). Another recently published study [21] described that artefacts were more pronounced in synthetic T2W FLAIR compared to conventional MR images.

In contrast to previous studies where synthetic FLAIR images suffered particularly from a perceived lower signal-to-noise-ratio and inferior overall image quality compared to conventional FLAIR [6, 8, 22], in this study a higher signal-to-noise ratio was observed in synthetic FLAIR images but also in synthetic T2W images. This can be explained by the larger voxel size used in the synthetic images compared to the conventional, even if the impact of reconstruction filters and parallel reconstruction, for example, remains difficult to evaluate [23]. The differences in measured lesion-to-white matter contrast did not obviously affect diagnostic performance.

Considering that this and earlier studies show variations in inter- and intra-observer agreement, the importance/relevance of absolute numbers may have to be put into perspective. In making the differential diagnosis of MS vs. non-MS, categorising lesion numbers according to the McDonald criteria [13] for instance is a useful tool, but in follow-up examinations the appearance of any single lesion can be of importance. Studies on this issue using synthetic MR are lacking so far. In a clinical context, though, the availability of prior examinations for comparison may facilitate the image interpretation.

Though quantitative MRI with reconstruction of synthetic images performed inferiorly in terms of overall inter- and intra-observer agreement quality and could not acquire 3D isotropic high-resolution images, there are several potential benefits of this approach. A major advantage of quantitative/synthetic MRI is the simultaneous acquisition of tissue parameters that can be used for automated calculation of tissue maps, tissue analysis and volume assessment [12, 24–29] and therefore may be a promising future alternative to evaluate focal and diffuse disease compared to assessment of focal lesions only. Furthermore, the imaging data are potentially scanner independent and scan times can be shortened. The total acquisition time for the conventional axial series (T1W, T2W and FLAIR) was in this study 18:30 (min:s) while the scan time for QRAPMASTER was 6:27.

This study has some limitations. There is a difference in experience between the two observers in terms of professionally active working years, though in our work the inter-observer variation was not higher than the intra-observer variation. The possibility of calculating other images than T1W, T2W and FLAIR to be used in the evaluation, such as IR T1W and double IR images, was not included in the study. In this study the 2D synthetic MR images were compared to corresponding 2D conventional MRI images even though 3D isotropic high-resolution imaging nowadays is an available alternative (3D was not available on the system when the study started) and is becoming more common in clinical routine. The use of 3-mm-thick slices, which was applied for both the synthetic and conventional images, implies a risk of missing small lesions because of partial volume effects compared to isotropic 3D acquisitions. The limitation of axial imaging may impede the evaluation of lesions in the corpus callosum and adjacent centrum semiovale. The in-plane resolution in the synthetic images was lower compared to the conventional images. The choice of resolution in the synthetic images was a compromise of scanning time, slice thickness and whole brain coverage. Another limitation is the variable existence of MS lesions in the study population, from a single or few lesions to multiple, in some cases nearly confluating, lesions, making counting lesions difficult at times. Studies comparing the diagnostic performance of synthetic MRI in 1.5 and 3 T may be needed as detection of MS lesions has been shown to be superior in 3 T, at least in conventional imaging [30], and significant differences in tissue segmentation between 1.5 and 3 T have been shown [28].

In conclusion, synthetic MR images have the potential to be used in the assessment of MS dissemination in space despite a slightly lower inter- and intra-observer agreement compared to conventional MR images. Studies evaluating the impact of those differences on clinical management and synthetic MR in the assessment of dissemination of MS lesions over time remain to be performed.

## Abbreviations

DIS: Dissemination in space
DIT: Dissemination in time
EDSS: Expanded disability status scale
FLAIR: Fluid attenuated inversion recovery
IR: Inversion recovery
MS: Multiple sclerosis
PACS: Picture-archiving and communication system
PD: Proton density
qMRI: Quantitative magnetic resonance imaging
TD: Delay time
TE: Echo time
TR: Repetition time
TI: Inversion time

## References

[1] Warntjes JB, Leinhard OD, West J, Lundberg P (2008). Rapid magnetic resonance quantification on the brain: Optimization for clinical usage. Magn Reson Med.

[2] Ma D, Gulani V, Seiberlich N (2013). Magnetic resonance fingerprinting. Nature.

[3] Ehses P, Seiberlich N, Ma D et al (2012) IR TrueFISP with a golden-ratio-based radial readout: Fast quantification of T(1) , T(2) , and proton density. Magn Reson Med 10.1002/mrm.24225 10.1002/mrm.24225 22378141

[4] Newbould RD, Skare ST, Alley MT, Gold GE, Bammer R (2010). Three-dimensional T(1), T(2) and proton density mapping with inversion recovery balanced SSFP. Magn Reson Imaging.

[5] Hagiwara A, Warntjes M, Hori M et al (2017) SyMRI of the brain: Rapid quantification of relaxation rates and proton density, with synthetic MRI, automatic brain segmentation, and myelin measurement. Investig Radiol. 10.1097/rli.0000000000000365 10.1097/RLI.0000000000000365 PMC5596834 28257339

[6] Granberg T, Uppman M, Hashim F et al (2016) Clinical feasibility of synthetic mri in multiple sclerosis: A diagnostic and volumetric validation study. AJNR Am J Neuroradiol. 10.3174/ajnr.A4665 10.3174/ajnr.A4665 PMC7963550 26797137

[7] Hagiwara A, Hori M, Suzuki M (2016). Contrast-enhanced synthetic MRI for the detection of brain metastases. Acta Radiol Open.

[8] Blystad I, Warntjes J, Smedby O, Landtblom AM, Lundberg P, Larsson EM (2012) Synthetic MRI of the brain in a clinical setting. Acta Radiol. 10.1258/ar.2012.120195 10.1258/ar.2012.120195 23024181

[9] Vagberg M, Axelsson M, Birgander R et al (2016) Guidelines for the use of magnetic resonance imaging in diagnosing and monitoring the treatment of multiple sclerosis: recommendations of the Swedish Multiple Sclerosis Association and the Swedish Neuroradiological Society. Acta Neurol Scand. 10.1111/ane.12667 10.1111/ane.12667 PMC5157754 27558404

[10] Lovblad KO, Anzalone N, Dorfler A (2010). MR imaging in multiple sclerosis: review and recommendations for current practice. AJNR Am J Neuroradiol.

[11] Rovira A, Wattjes MP, Tintore M (2015). Evidence-based guidelines: MAGNIMS consensus guidelines on the use of MRI in multiple sclerosis-clinical implementation in the diagnostic process. Nat Rev Neurol.

[12] Warntjes JB, Tisell A, Landtblom AM, Lundberg P (2014) Effects of gadolinium contrast agent administration on automatic brain tissue classification of patients with multiple sclerosis. AJNR Am J Neuroradiol. 10.3174/ajnr.A3890 10.3174/ajnr.A3890 PMC7966571 24699093

[13] Polman CH, Reingold SC, Banwell B (2011). Diagnostic criteria for multiple sclerosis: 2010 revisions to the McDonald criteria. Ann Neurol.

[14] Krauss W, Gunnarsson M, Andersson T, Thunberg P (2015). Accuracy and reproducibility of a quantitative magnetic resonance imaging method for concurrent measurements of tissue relaxation times and proton density. Magn Reson Imaging.

[15] Warntjes JB, Dahlqvist O, Lundberg P (2007). Novel method for rapid, simultaneous T1, T*2, and proton density quantification. Magn Reson Med.

[16] Altman DG, Bland MJ (1986). Statistical methods for assessing agreement between two methods of clinical measurement. Lancet.

[17] Bland JM, Altman DG (1986). Statistical methods for assessing agreement between two methods of clinical measurement. Lancet.

[18] Barkhof F, Filippi M, van Waesberghe JH, Campi A, Miller DH, Ader HJ (1999). Interobserver agreement for diagnostic MRI criteria in suspected multiple sclerosis. Neuroradiology.

[19] Zipoli V, Portaccio E, Siracusa G, Pracucci G, Sorbi S, Amato MP (2003). Interobserver agreement on Poser's and the new McDonald's diagnostic criteria for multiple sclerosis. Mult Scler.

[20] Korteweg T, Uitdehaag BM, Knol DL (2007). Interobserver agreement on the radiological criteria of the International Panel on the diagnosis of multiple sclerosis. Eur Radiol.

[21] Tanenbaum LN, Tsiouris AJ, Johnson AN (2017). Synthetic MRI for clinical neuroimaging: Results of the magnetic resonance image compilation (MAGiC) prospective, multicenter, multireader trial. AJNR Am J Neuroradiol.

[22] Betts AM, Leach JL, Jones BV, Zhang B, Serai S (2016). Brain imaging with synthetic MR in children: clinical quality assessment. Neuroradiology.

[23] Dietrich O, Raya JG, Reeder SB, Reiser MF, Schoenberg SO (2007). Measurement of signal-to-noise ratios in MR images: influence of multichannel coils, parallel imaging, and reconstruction filters. J Magn Reson Imaging.

[24] Warntjes JB, Engstrom M, Tisell A, Lundberg P (2013). Brain characterization using normalized quantitative magnetic resonance imaging. PLoS One.

[25] Hagiwara A, Hori M, Yokoyama K et al (2016) Utility of a multiparametric quantitative MRI model that assesses myelin and edema for evaluating plaques, periplaque white matter, and normal-appearing white matter in patients with multiple sclerosis: A feasibility study. AJNR Am J Neuroradiol. 10.3174/ajnr.A4977 10.3174/ajnr.A4977 PMC7963826 27789453

[26] Blystad I, Hakansson I, Tisell A (2016). Quantitative MRI for analysis of active multiple sclerosis lesions without gadolinium-based contrast agent. AJNR Am J Neuroradiol.

[27] West J, Aalto A, Tisell A (2014). Normal appearing and diffusely abnormal white matter in patients with multiple sclerosis assessed with quantitative MR. PLoS One.

[28] West J, Blystad I, Engstrom M, Warntjes JB, Lundberg P (2013). Application of quantitative MRI for brain tissue segmentation at 1.5 T and 3.0 T field strengths. PLoS One.

[29] West J, Warntjes JB, Lundberg P (2012). Novel whole brain segmentation and volume estimation using quantitative MRI. Eur Radiol.

[30] Stankiewicz JM, Glanz BI, Healy BC (2011). Brain MRI lesion load at 1.5T and 3T versus clinical status in multiple sclerosis. J Neuroimaging.

